# Synthesis of a Biodegradable Polymer of Poly (Sodium Alginate/Ethyl Acrylate)

**DOI:** 10.3390/polym13040504

**Published:** 2021-02-07

**Authors:** Cynthia G. Flores-Hernández, Maria de los Angeles Cornejo-Villegas, Abigail Moreno-Martell, Alicia Del Real

**Affiliations:** 1 Tecnológico Nacional de México/Instituto Tecnológico de Querétaro, Av. Tecnológico s/n Esq. Gral. Mariano Escobedo, Col. Centro Histórico, Santiago de Querétaro C.P. 76000, Querétaro, Mexico; cynthiagraciela84@gmail.com; 2 Laboratorio de Procesos de Transformación y Tecnologías Emergentes de Alimentos, Departamento de Ingeniería y Tecnología, FES-Cuautitlán, Universidad Nacional Autónoma de México, Cuautitlán Izcalli C.P. 76000, Edo. de Mexico, Mexico; angiecornejo@unam.mx; 3 Departamento de Ingeniería Molecular de Materiales, Centro de Física Aplicada y Tecnología Avanzada, Universidad Nacional Autónoma de México, Campus Juriquilla, Querétaro C.P. 76230, Qro., Mexico; morenomartell@gmail.com

**Keywords:** sodium alginate, graft copolymerization, biodegradable polymer

## Abstract

The objective of the study was to obtain a new biodegradable graft polymer by performing two chemical processes: first, a transesterification reaction between carboxylic acid’s salt and ethyl acrylate’s ester, followed by polymerization of the vinyl group from the ethyl acrylate monomer via free radicals. The copolymer’s FTIR shows an absence of ethyl bands, while the characteristic band of pyranose is maintained, which confirms the monomer’s graft. TGA analysis shows that sodium alginate had three decomposition temperatures: 103 °C due to dehydration, 212 °C associated with the destruction of glycosidic bonds, and 426 °C due to conversion of alginate into Na_2_CO_3_. The copolymer presents four processes at different temperatures, i.e., evaporation of alcohol at 65 °C, decomposition of ungrafted alginate at 220 °C, copolymer decomposition at 298 °C, and degradation of fragments into carbonate at 423 °C. The evaluation of the action of fungal growth on the copolymer was higher than 50%, which means it is an excellent material to be biodegraded.

## 1. Introduction

Polysaccharides are biodegradable natural polymers made up of monosaccharides linked by glucoside bonds [1]. Examples are starch, cellulose, glycogen, alginic acid, agar, carrageenan, chitosan, xanthan gum, dextran, pectin, and others [2,3]. Numerous studies have focused on investigating the modification of these polysaccharides to improve their functional properties. The modifications used are either from the mixture of polysaccharides with synthetic polymers, the in-situ polymerization of a monomer giving rise to interpreted networks (IPN), or the grafting of a monomer on the polysaccharide chain with its subsequent polymerization, resulting in graft cross-linking.

Sodium alginate is a linear polysaccharide derived from alginic acid composed of 1,4-β-d-mannuronic acid (M) and α-1-guluronic acid (G) [4,5]. Alginate has an abundance of free hydroxyl and carboxyl groups distributed along the main chain; these two functional groups have the capacity for chemical modification such as oxidation, sulphation, esterification, or grafting methods [6,7,8,9].

In this context, acrylic polymers are an option to modify polysaccharides, as is the case with sodium alginate [10,11,12]. For example, poly(ethyl acrylate) is used to improve the physical properties of synthetic polymers and to improve the properties of polysaccharides, such as cellulose fibers from wood pulp and other renewable materials such as tamarind seeds [13,14].

Hydrogels can be synthesized from sodium alginate with acrylic polymers or copolymers, and with other compounds such as acrylamide to create an interpenetrating network (IPN) between the polymers based on electrostatic phenomena [15]. In addition, there are investigations in which a polymerization of sodium alginate (SA) and an acrylic monomer is carried out, using in most cases a crosslinker such as N, N-methylene-bis-acrylamide (MBAA) or ethylene glycol dimethacrylate (EGDMA); to obtain hydrogels with different applications [16,17,18].

There are even some works where a cross-linker is not used to obtain a graft polymer, as in the case of copolymers of sodium alginate (NaAlg) with itaconic acid (IA). These were prepared in an aqueous solution using benzoyl peroxide (BPO) or nitrate of cerium (IV) ammonium/nitric acid (CAN/HNO_3_) as initiators for a cross-linked polymerization [19,20]. On the other hand, Sahera et al. in 2013 [21] studied hydrophilic hydrogels based on poly(acrylic acid) as a synthetic polymer and sodium alginates as a natural polymer (AG). The cross-linking was prepared by gamma irradiation formation of interpenetrating AAc/AG network structures [21]. In addition, other hydrophilic hydrogels based on poly(acrylic acid) and sodium alginates were prepared by gamma irradiation with different applications [10,22].

However, the only publication found with ethyl acrylate to synthesize a graft copolymer with sodium alginate used ceric ammonium nitrate as an initiator [23]. Therefore, although there are different research studies on the use of sodium alginate with acrylic polymers and the grafting method, each method, initiator, and monomer offer different structures and, therefore, the possibility to diversify the properties of the copolymer. Thus, the aim of this research is to obtain a new biodegradable graft polymer by performing two chemical processes: first, a transesterification reaction between carboxylic acid salt and ethyl acrylate ester, followed by free radical polymerization of the vinyl group of the ethyl acrylate (EA) monomer, and as initiator α,α,′-Azoisobutyronitrile; (AIBN) and a cross-linking agent was not used to carry out the polymerization.

## 2. Materials and Methods

For the synthesis of the copolymer we used sodium alginate (sodium polymanuronate) (Golden Bell^®^ CA, USA) USP grade, ethyl acrylate (EA; Sigma-Aldrich, St. Louis, MO, USA), and as initiator α,α,′-Azoisobutyronitrile (AIBN; Sigma-Aldrich, St. Louis, MO, USA) with a purity of 98%.

For biodegradation tests, we used as a culture medium dextrose and potato agar (DPA BD Bioxon, Edo. de Mex., México), bacteriological agar (BD Bioxon, Edo. de Mex., México), trisodium citrate pentahydrate (Merck, Edo. de Mex., México), monophasic anhydrous potassium phosphate (Merck, Edo. de Mex., México), monobasic anhydrous potassium phosphate (J,T, Baker, Phillipsburg, NJ, USA), copper (II) pentahydrate sulfate (Sigma-Aldrich, St. Louis, MO, USA), manganese sulfate monohydrate, anhydrous orthoboric acid (JT Baker, Phillipsburg, NJ, USA), chloroform (Sigma-Aldrich, St. Louis, MO, USA), magnesium sulfate heptahydrate (Merck), sucrose (Sigma-Aldrich, St. Louis, MO, USA) and glycerol 99.5% purity (Sigma-Aldrich, St. Louis, MO, USA). 

The fungus Alternaria spp. isolated in our laboratory was used for the biodegradation. 

### Synthesis

For the preparation of the poly graft copolymer (AlgNa/EA), 7.5 g of sodium alginate was dissolved in 300 mL of distilled water at 95 °C with mechanical stirring for 3 h, allowed to cool to room temperature. Then, 5 g of ethyl acrylate was dissolved with mechanical stirring, and the solution was brought to a temperature of 75 °C; nitrogen was bubbled to remove dissolved oxygen, and then 0.040 g of AIBN initiator was added. The polymerization was carried out for 6 h. The resulting material was washed three times with abundant acetone to remove residual homopolymer and to precipitate the copolymer. Subsequently, the precipitate was re-dissolved in water and dried in a petri dish, and a film of a thickness of 0.125 ± 0.003 mm was obtained. Of all the formulations made during research, this article presents the one where the results of the graft became most evident. In order to have a comparison parameter, poly(ethyl acrylate) (PEA) was synthesized under the same conditions as for the copolymer in the absence of sodium alginate (AlgNa).

## 3. Characterization

### 3.1. FTIR Spectroscopy

PEA, AlgNa, and poly(AlgNa/EA) films were analyzed on a spectrometer Bruker vector 33 ATR-FTIR Bruker Spectrometer I18500 PS15 Billerica, MA, USA, Power Supply 115/230 V in the range of 4000 to 400 cm^−1^; for each test, 32 scans were applied with smoothing. With this technique, the functional groups were determined for each compound, as well as the vibration of the group that forms the graft bond.

### 3.2. Differential Scanning Calorimetry (DSC)

The glass transition temperature (T_g_) was characterized by PEA, AlgNa, and poly(AlgNa/AE) by the differential scanning calorimetry technique in a TA INSTRUMENTS DSC Thermo DSC Q 100 Thermo Model 970701.901 DSC System With Thermo DSC Refrigerated Cooling System Thermo Model FC100AX0TA Thermo DSC Thermo Differential Scanning Calorimeter, New Castle, DE, USA., Here, 8 mg of the sample was subjected to heating in a range of −50 to 230 °C, with a heating rate of 10 °C/min in a nitrogen atmosphere.

### 3.3. Thermogravimetric Analyses (TGA)

The thermogravimetric analyses were carried out with a TA Instruments Q5000IR TGA MS temperature range ambient to 1200 °C, New Castle, DE, USA, Thermal Analysis System series. Samples of approximately 5 mg were subjected to heating at a rate of 10 °C/min under a nitrogen atmosphere ranging from 25 to 600 °C. With this technique, the loss of mass in each thermal transition, the decomposition of the copolymer, and the thermal stability for each compound was determined.

### 3.4. Tensile Test

Tensile tests were performed on an INSTRON model 5500R Norwood, MA, USA, with a load cell of 0.5 kN, and at a speed of 1 mm/min. Five specimens were made, on average, per sample, in accordance with ASTM D-1708-02a.

### 3.5. Evaluation of the Action of a Microorganism of Plastics Biodeterioration

The fungal-growth test is suitable for the assessment of the inherent resistance of plastics to fungal attack in the absence of other organic matter. Test specimens were exposed to a mixed suspension of fungal spores in the presence of a humidity ≥ 95% relative humidity. After the limited nutrients from the spore itself are depleted through the formation of a germination tube, the fungi can only grow at the expense of the material of the test specimens. If the specimens contain no nutritive component, the fungi cannot develop mycelia, and there will be no deterioration of the plastic.

Deterioration of films tests was carried out in a solid medium, using the petri dish screen method and the method described in UNE-EN ISO 846:1997, “Evaluation of the action of microorganisms” [24]. This technique uses visual inspection to evaluate the lability to fungal attack, and based on the inspection, a numeric level is assigned as follows: 0 for no growth apparent under the microscope; 1 for no growth visible to the naked eye, but visible under the microscope; 2 for growth visible to the naked eye, covering up to 25% of the test surface; 3 for growth visible to the naked eye, covering up to 50% of the test surface; 4 for considerable growth, covering more than 50% of the test surface; and 5 for heavy growth, covering the entire test surface [24]. In moisture chambers, UV-sterilized 1 × 1 cm^2^ copolymer films were placed separately on an object carrier that was inoculated with the fungus Alternaria spp., in one of the film corners. The moisture chambers were incubated at 30 °C for two months. The results were observed weekly with the scanning electron microscope JEOL-JSM-6060LV Tokyo, Japan, at a voltage of 20 KV.

## 4. Results and discussion

### 4.1. Fourier-Transform Infrared Spectroscopy (FTIR)

**Figure 1:**
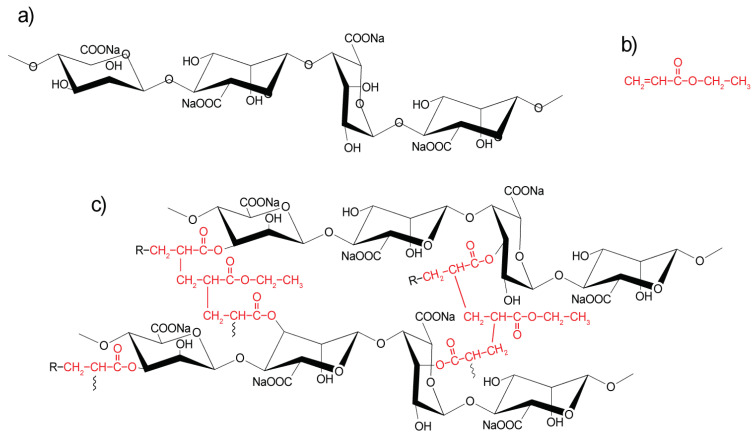
Chemical structures of (**a**) AlgNa, **(b**) PEA, and (**c**) poly(AlgNa/EA).

**Figure 2:**
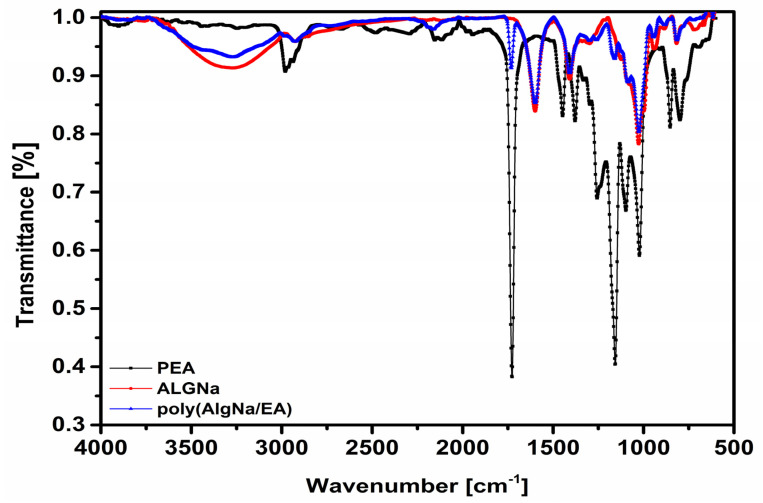
FTIR-ATR spectrum (PEA), (AlgNa), and poly (AlgNa/EA).

The FTIR analysis was used to investigate the molecular interactions (Figure 1) between AlgNa, PEA, and poly(AlgNa/EA). Figure 2 also shows the spectra, and Table 1 summarizes the absorption band assignments of these materials. Figure 2 also shows the spectra of AlgNa and poly(AlgNa/EA), where broad absorption bands between 3650 and 3000 cm^−1^ can be observed; the lowest points of each band are at 3275 and 3271 cm^−1^, respectively. These bands can be attributed to the stretching vibrations of the O–H groups present in polysaccharides and hydrogen-bonded H_2_O molecules [25]. The aforementioned spectra each contain also a band at 2925 cm^−1^, attributed to C–H stretching from the pyranose ring of the polysaccharide [25,26].

The AlgNa spectrum Figure 2 contains a set of bands that can each be attributed to a certain vibration from C–O groups. At 1598 cm^−1^, it corresponds to the asymmetric stretching in COONa, at 1409 cm^−1^ to the symmetric stretching of COO–, at 1027 and 945 cm^−1^ to symmetric stretching in the pyranose ring. The 883 cm^−1^ band is attributed to C–O–C symmetric stretching vibration present in 1,4 glycosidic links, while 817 cm^−1^ is attributed to flexion vibration in guluronic and mannuronic acid units, see Table 1.

All the bands are present in the copolymer, albeit being displaced in some cases, such as 1027 cm^−1^ moving to 1026 cm^−1^, 945 cm^−1^ to 944 cm^−1^, 883 cm^−1^ to 881 cm^−1^, and 817 cm^−1^ to 815 cm^−1^, which means the structure of the alginate is preserved. The band corresponding to the symmetric stretching C–O of the COO^−^ group presents the biggest displacement (1409 cm^−1^ to 1386 cm^−1^) due to the esterification reaction occurring in that group see Table 1.

Table 1 for the PEA compound shows the characteristic of vibration of the C–H bond of the ethyl group the symmetric stretching CH_3_ is located at 2979 cm^−1^, the asymmetric stretching CH_3_ is located at 2964 cm^−1^, and the asymmetric stretching CH_2_ of ethyl ester is at 2937 cm^−1^. The 1469 cm^−1^, 1446 cm^−1^, 1379 cm^−1^, 1332 cm^−1^, 852 cm^−1^, and 761 cm^−1^ bands are attributed to asymmetric stretching CH_2_, asymmetric stretching CH_3_, asymmetric stretching CH_3_, twisting CH_2_, stretching C–C, and rocking CH_2_ of ethyl groups, respectively [25]; these bands are not found in poly(AlgNa/EA), and the lack thereof is attributed to transesterification, where the PEA’s ethyl groups are lost. The presence of the signal located at 2190 cm^−1^ corresponds to the triple bond of the C–N nitrile group from the initiator AIBN that is anchored to the chain during polymerization.

Table 1 shows the characteristic vibrations of the C-H bond of the ethyl group, the symmetric stretching CH3 is located at 2979 cm^−1^, the asymmetric stretching CH3 is located at 2964 cm^−1^, and the asymmetric stretching CH2 of ethyl ester is at 2937 cm^−1^. Table 1 for PEA shows, at 1726 cm^−1^ due to the O=C stretching vibration of the ester group [25], at 1469 cm^−1^ due to CH_2_ deformation vibration of ethyl ester, at 1257 cm^−1^ and 1157 cm^−1^ correspond to the asymmetric and symmetric O=C stretching of the R–CO–OR group [14,25], at 1097 cm^−1^ due to the stretching the C–O–C bond of the saturated esters. [13,27], and at 1022 cm^−1^ due to the stretching of the ester group [25,28,29]. 

None of the bands related to the ethyl group are present in poly(AlgNa/EA); the other bands exhibit displacements, such as from 1257 cm^−1^ to 1261 cm^−1^ and from 1157 cm^−1^ to 1161 cm^−1^ due to C=O deriving from the ester group, and from 1022 cm^−1^ to 1026 cm^−1^ due to C–O stretching of carbohydrate and ester group. These displacements at longer wavelengths can be attributed to the restriction of movement generated by the graft during polymerization.

### 4.2. Thermogravimetric Analysis (TGA)

**Figure 3:**
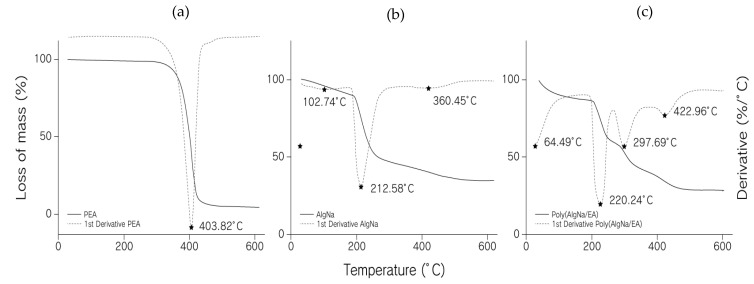
TGA thermograms of (**a**) PEA, (**b**) AlgNa, and (**c**) poly(AlgNa/EA) weight (%) vs. temperature and first derivate weight (%) and identification of the changes present in each graph.

Thermogravimetric analysis (TGA) is an experimental technique in which the weight or, strictly speaking, the mass of a sample is measured as a function of sample temperature or time. Mass changes occur when the sample loses material in one of several different ways or reacts with the surrounding atmosphere. This produces steps in the TGA curve or peaks in the differential thermal gravimetric (DTG) curve [30]. Figure 3 illustrates TGA and differential TGA (DTG) curves of (a) PEA, (b) AlgNa, and (c) poly(AlgNa/EA). Figure 3a, thermogram PEA showed only one decomposition stage at 400 °C with a weight loss of 96%, implying the total degradation of the polymer [31].

In Figure 3b, the thermogram for AlgNa shows 3 thermogravimetric effects, with the first being a 10% mass loss at 103 °C due to the dehydration of water linked by hydrogen bridges (adsorbed). The energy of bonding between water molecules and the sorption sites is higher than the energy that holds the molecules of pure water [32]. The second weight loss of about 41% was observed at 212 °C; this weight loss is typically associated with the destruction of glycosidic bonds [9,33] and corresponds to alginate fragmentation due to chain breakage. The last thermogravimetric effect occurred at 426 °C with a weight loss of 12.4%, where the fragments and monomeric units of the alginate are converted into Na_2_CO_3_ [9,33].

In Figure 3c, poly(AlgNa/EA) four thermal processes are observed, at 65 °C there is the evaporation of an alcohol mixture (generated in the transesterification) and water (bound to alginate) with a weight loss of 13%, at 220 °C it is attributed to the decomposition of sodium alginate, with a weight loss of 25%. At 298 °C, it is attributed to the decomposition of the copolymer, with a weight loss of 16%. Therefore, it is confirmed that the synthesis of graft copolymerization between alginate and ethyl acrylate has higher thermal stability than the unmodified polysaccharide. Finally, the peak present at 423 °C with 18% weight loss is attributed to the degradation of fragments and monomer unit’s conversion into carbonate.

### 4.3. Differential Scanning Calorimetry (DSC)

DSC measures the flows of energy into or out of a material in response to changes in temperature, which is associated with heat-induced macromolecular transitions [34]. Glass transition is inherent of amorphous polymer materials, and these occur between the glassy and high-elastic states. Below T_g_, the polymer is in a glass state, in which the molecular chain and the chain segment cannot move; above T_g_, the polymer structure is mobile, and large-scale molecular motion is possible [35]. The glass transition is not considered a first-order phase transition, but a kinetic phenomenon, or a second-order transition [36]. The relaxation enthalpy of an amorphous polymer is a phenomenon that arises from the fact that these materials are in a non-equilibrium state when maintained at temperatures below their T_g_ [37].

**Figure 4:**
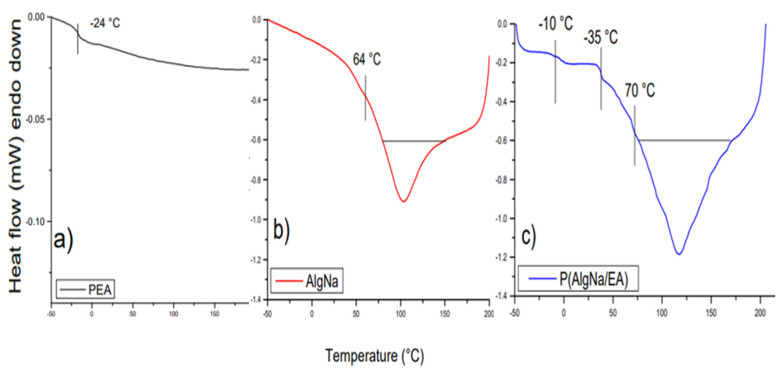
DSC thermograms of (**a**) PEA, (**b**) AlgNa, and (**c**) poly(AlgNa/EA).

Figure 4 shows DSC thermograms curves for (a) PEA, (b) AlgNa, and (c) poly(AlgNa/EA). To determine glass transition temperatures, the point where the slope of the curve changes slightly was found, and the corresponding enthalpies were calculated by measuring the area in the curve delimited by a straight line [38]. In Figure 4a, the glass transition temperature of the PEA was −24 °C. In Figure 4b, sodium alginate’s T_g_ is 64 °C; there is a wide endothermic peak, which starts around 80 °C and ends at 150 °C, and ∆H = 10 Jg^−1^, where the area under the endotherm associated with T_g_ is defined as enthalpy recovery or relaxation and evaporation of water linked. Several authors generally attribute this process to gelatinization [39,40]. However, we consider that claim to be inaccurate, and would not attribute it to gelatinization but relaxation enthalpy [38]. On the other hand, the TGA analysis of AlgNa shows a loss of mass in this interval, which could also be due to the evaporation enthalpy of water molecules linked to the polysaccharide corresponding to non-freezing bound water [32]. Glass transition is often referred to as a second-order phase transition that occurs without the release or absorption of latent heat. However, due to the non-equilibrium nature of the glassy states, the glass transition is preferably called a state transition, rather than a phase transition [41].

In the curve of poly(AlgNa/EA)’s thermogram in Figure 4c, it is possible to observe three transition points, with the first point resting at T_g_ = −10 °C, corresponding to the partially modified alginate. The next point lies at T_g_ = 35 °C, which is attributed to the non−reacting alginate. The third point at T_g_ = 70 °C presents a great endothermic trough from 75 to 170 °C (∆H = 28 Jg^−1^), attributed to poly(AlgNa/EA). By comparing the enthalpy values of the copolymer and sodium alginate, it is apparent that it is greater for the copolymer, which indicates there is a higher interaction between the copolymer’s molecule, which requires more energy to relax.

### 4.4. Tensile Test

The tensile strength and the strain percentage are important parameters for the mechanical properties of synthesized films, which depend on the microstructural characteristics of the material. The tensile strength is the maximum point in the strain–stress curve, and the percentage of elongation (% E) is the maximum elongation of the film before the break [24]. Table 2 shows the elastic modulus, strain percentage, and tensile strength of (a) AlgNa, (b) PEA, and (c) poly(AlgNa/EA).

According to the results obtained in the tensile tests, the grafting of ethyl polyacrylate in sodium alginate caused an increase in elongation of 22% in the copolymer concerning pure AlgNa. This behavior can be attributed to the low glass transition temperature (−24 °C) presented by the PEA, which causes a change in flexibility to AlgNa. In addition, an increase in the value of tensile strength in poly(AlgNa/AE) of 97.92 MPa for polysaccharides (93.26 MPa) was obtained. This increase could be associated with the possible existence of a cross-linking between the polymers, generated by the interaction between the carboxyl groups of the synthetic polymer with the hydroxyl groups of the polysaccharide, which makes the copolymer a more resistant material and therefore, more strength is required for its fracture. However, the elastic modulus value shows a decrease in poly(AlgNa/EA) (2436 MPa) with respect to AlgNa (3226 MPa) as the concentration of the PEA increases.

### 4.5. Deterioration of Plastics by Scanning Electron Microscopy

**Figure 5:**
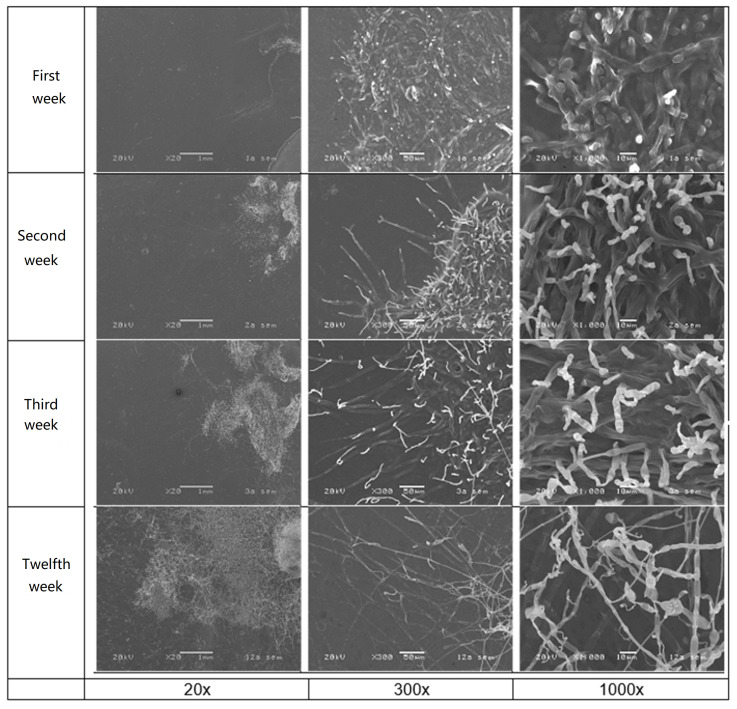
SEM images of fungal growth on poly(AlgNa/EA) copolymer film at different magnifications 20×, 300×, and 1000× as a function of time in weeks.

The determination of the biodeterioration of plastics due to the action of bacteria and fungi can be carried out through international standards, in which the type and extent of the biodeterioration can be determined by visual examination, by mass variations, and by variations in other physical properties. The tests may apply to all products made of plastic materials that have a flat surface and can be easily cleaned [24]. Figure 5 shows an SEM image of the growth of fungus *Alternaria* sp. over the copolymer poly(AlgNa/EA) film as a function of time; the growth of the fungus on the polymer membranes was inspected every week for two months (12 weeks). In Figure 5, only weeks 1, 2, 3, and 12 of the study are shown because the biodeterioration changes observed in those weeks were deemed the most representative. The microscopic characteristics of the fungus *Alternaria* spp. are hyphae, spores, and conidia.

In the first week, it was detected that the fungus was incubated satisfactorily; its growth began, and it can be seen how the hyphae are anchored or attached to the film. In the second week, the increase in the number of hyphae on the films was observed, which are identified with a structure with better definition, maturity attributed to the incubation time. In the third week, the amount of the fungus has increased due to the enzymatic action that was being generated between the fungus and the copolymer film. Figure 5 shows the completely mature hyphae and with a better definition than in the previous weeks. Finally, in 12 weeks the image from SEM shows that the plastic film has been invaded by fungi. As a result, the fungus has matured at the expense of nutrients from the copolymer; this is indicative of the degradation of the plastic film. The fungal growth on the surface was higher than 50%, which means it is an excellent material to be degraded.

## 5. Conclusions

All the bands from sodium alginate are present in the copolymer, such as the characteristic bands of the pyranose ring, albeit being displaced in some cases, which means the structure of the alginate is preserved. The band COO^−^ group presents the biggest displacement. Furthermore, none of the bands from the ethyl group of ethyl acrylate’s monomer is present in the copolymer, which demonstrates transesterification. The C=O bands deriving from the acrylate monomer suffer a displacement when trans-esterified. These displacements at longer wavelengths can be attributed to the restriction of movement generated by the graft during polymerization.

The copolymer’s TGA shows the presence of a modified part of the alginate and the grafted copolymer, as well as the absence of PEA. In the DSC there are 3 observable transition points: The first two are attributed to T_g_ values for the modified alginate and alginate, both partially miscible, which makes transitions displace. The third transition point is attributed to poly(AlgNa/EA). The increase of relaxation enthalpy for the copolymer is due mainly to the cross-links of chains between alginate and monomer, which generates an impediment of copolymer molecules to relax.

According to the international standard ISO 846 [24], the fungal growth on the surface of copolymers was higher than 50%, which means it is a good material to be biodegraded. Finally, these results, in which free radical graft with carbohydrates and acrylic monomers is performed, allow us to develop new and environmentally friendly materials.

## Figures and Tables

**Table 1:** FTIR absorption bands (cm^−1^) for (AlgNa), (PEA), and poly (AlgNa/EA).

AlgNa	PEA	poly (AlgNa/EA)	Assignment
3275		3271	O–H stretching
2925		2925	C–H stretching of the pyranose ring
	2979	--------	Symmetric stretching C–H of CH_3_ of Ethyl ester
	2964	--------	Asymmetric stretching C–H of CH_3_ of Ethyl ester
	2937	--------	Asymmetric stretching C–H of CH_2_ of Ethyl ester
	2190	2190	Stretching C☰N nitrile of initiator
	1726	1732	stretching O=C of ester
1598		1598	Asymmetric stretching C–O of COONa
	1469	------	Asymmetric stretching C–H of CH_2_ of ethyl Deformation vibration of the CH_2_ ethyl ester
	1446	------	Asymmetric stretching CH_3_ of ethyl Symmetric stretching CH_2_ esters acrylic
1409		1386	Symmetric stretching C–O of the COO^−^
	1379	------	Symmetric stretching CH_3_ of Ethyl
	1332	-------	Twisting CH_2_ of the ethyl group
	1257	1261	Asymmetric stretching O=C of R–CO–OR
	1157	1161	Symmetric stretching C=O of R–CO–OR
	1097	------	Stretching of C–O–C of saturated esters
1027	1022	1026	Stretching C–O of C–O–C carbohydrate and ester
945		944	Symmetric stretching C–O and/or symmetric stretching C–C–H pyranose ring
883		881	Symmetric stretching vibration of C–O–C of 1,4 glycosidic links, characteristic of polysaccharides structure ring
	852	------	Stretching C–C of ethyl
817		815	δ C–O–C guluronic and mannuronic acid unit
	761	------	CH_2_ rocking

**Table 2:** Elastic modulus of AlgNa, PEA, and poly(AlgNa/EA).

Nomenclature	Elastic Modulus (MPa)	Tensile Strength (MPa)
AlgNa	3226	93.26
PEA	0.006	0.585
Poly(AlgNa/EA)	2436	97.92
